# Molecular Epidemiology of HIV-1 Transmission in a Cohort of HIV-1 Concordant Heterosexual Couples from Dakar, Senegal

**DOI:** 10.1371/journal.pone.0037402

**Published:** 2012-05-17

**Authors:** Wim Jennes, Jordan K. Kyongo, Evelyn Vanhommerig, Makhtar Camara, Sandra Coppens, Moussa Seydi, Souleymane Mboup, Leo Heyndrickx, Luc Kestens

**Affiliations:** 1 Laboratory of Immunology, Department of Biomedical Sciences, Institute of Tropical Medicine, Antwerp, Belgium; 2 Laboratory of Immunology, Department of Bacteriology-Virology, Centre Hospitalier Universitaire Le Dantec, Cheikh Anta Diop University, Dakar, Senegal; 3 Laboratory of Virology, Department of Biomedical Sciences, Institute of Tropical Medicine, Antwerp, Belgium; 4 Department of Infectious Diseases, Centre Hospitalier Universitaire Fann, Cheikh Anta Diop University, Dakar, Senegal; University of California San Francisco, United States of America

## Abstract

**Background:**

A large number of HIV-1 infections in Africa occur in married couples. The predominant direction of intracouple transmission and the principal external origins of infection remain important issues of debate.

**Methods:**

We investigated HIV-1 transmission in 46 HIV-1 concordant positive couples from Dakar, Senegal. Intracouple transmission was confirmed by maximum-likelihood phylogenetic analysis and pairwise distance comparisons of HIV-1 *env* gp41 sequences from both partners. Standardized interview data were used to deduce the direction as well as the external sources of the intracouple transmissions.

**Results:**

Conservative molecular analyses showed linked viruses in 34 (74%) couples, unlinked viruses in 6 (13%) couples, and indeterminate results for 6 (13%) couples. The interview data corresponded completely with the molecular analyses: all linked couples reported internal transmission and all unlinked couples reported external sources of infection. The majority of linked couples (93%) reported the husband as internal source of infection. These husbands most frequently (82%) reported an occasional sexual relationship as external source of infection. Pairwise comparisons of the CD4 count, antiretroviral therapy status, and the proportion of gp41 ambiguous base pairs within transmission pairs correlated with the reported order of infection events.

**Conclusions:**

In this suburban Senegalese population, a majority of HIV-1 concordant couples showed linked HIV-1 transmission with the husband as likely index partner. Our data emphasize the risk of married women for acquiring HIV-1 as a result of the occasional sexual relationships of their husbands.

## Introduction

The HIV-1 epidemic in Sub-Saharan Africa is mainly driven by heterosexual transmission [1], [2]. Many individuals in stable relationships are infected [3], which has led to a growing interest in understanding the dynamics and risk factors of within-couple HIV-1 transmissions. Early studies observed that, in most cases, husbands acquire HIV-1 infection first from outside the marriage and then go on to infect their wives, and thus that risk factors for couples are actually those for the male partners [4]–[6]. Indeed, extramarital relationships of men are often culturally tolerated while women have difficulties in negotiating condom use during marital sex, together increasing the risk of HIV-1 infection for women during marriage [7], [8]. However, recent data have questioned these conclusions. Meta-analysis studies observed large proportions of HIV-1 discordant couples in African countries [9], with women as likely as men to be the HIV-1 positive partner in these couples [10]. Based on these data, it was estimated that the majority of HIV-1 infections in Africa occur within married couples, with comparable frequencies of male-to-female and female-to-male transmissions [11]. Thus, contrary to previous understanding, these data suggest that marriage also poses a considerable risk of HIV-1 infection for men.

A related topic under debate involves the role played by concurrent sexual relationships, i.e. having several sexual partners at the same time, in the African HIV-1 epidemic. It was postulated that a high frequency of concurrent sexual relationships results in an interlocking sexual network that could greatly facilitate the spread of HIV-1 [12]. Although this hypothesis is supported by circumstantial evidence [13]–[17], other studies failed to observe such associations [18]–[21]. A possible explanation for these opposite findings may lie in the uncertainty of the nature of the concurrent relationships that pose the greatest risk. For example, population levels of polygyny, a culturally embedded and institutionalized form of concurrency, were actually found to correlate inversely with HIV-1 prevalence [22]. Another crucial aspect lies in the way these associations are measured. Given that individuals with concurrent sexual relationships especially put their stable partners at increased risk of HIV-1 infection, these stable partners have to be included in the analyses [23].

Most studies of HIV-1 transmission in African married couples used prospectively followed cohorts of HIV-1 discordant couples. These studies may suffer from inclusion bias and changes in sexual behavior due to counseling and follow up. Studies of couples with an already HIV-1 concordant status do not have these problems, yet to date few studies have analyzed the patterns of HIV-1 transmission using such cohorts. In the present study, we set out to unravel intracouple HIV-1 transmission in a cohort of HIV-1 concordant positive couples from Dakar, Senegal. Couples with genetically linked viruses were first selected by phylogenetic analysis of HIV-1 *env* gp41 sequences from both partners. Standardized interview data from both partners reporting separately on the history of their HIV-1 infection were then used to analyze the direction of the intracouple transmission as well as the external sources of the infection. In this way, we found that a majority of HIV-1 concordant couples harbored linked HIV-1 sequences with the husband as the likely index partner in these couples.

## Materials and Methods

### Study outline

From May 2005 until February 2009, HIV patients consulting at the outpatient clinic of the Centre Hospitalier Universitaire Fann in Dakar, Senegal were invited to participate with their partners in a follow up study investigating correlates of protection against HIV. During the screening visit, participants donated blood samples for HIV testing and they were interviewed by a social assistant about socio-demographics and sexual behavior. Participants who tested HIV positive received individual counseling during which they were advised to disclose their status to their partner, and they were enrolled for clinical follow up with access to antiretroviral therapy if needed. Couples with an HIV discordant status were invited for follow-up every four months for a period of up to three years. Couples with an HIV concordant positive status were invited for one additional visit to the clinic four months after the screening visit. During every visit, the participants donated blood samples for laboratory testing and they were interviewed about socio-demographics and sexual behavior. All participants received safe sex counseling during every visit. The present study investigates intracouple transmission of all enrolled HIV-1 concordant couples. HIV-1 concordant couples with confirmed intracouple transmission will be used along with HIV-1 discordant couples in future studies of HIV protective immunity.

### Ethics statement

The study was approved by the Internal Review Board of the Institute of Tropical Medicine (Antwerp, Belgium), the Ethical Committee of the University Hospital of Antwerp (Antwerp, Belgium), and the Ethical Committee of the Senegalese Ministry of Health (Dakar, Senegal). All subjects gave written informed consent before enrolment.

### Laboratory methods

Whole blood was drawn in EDTA tubes (Becton Dickinson). Plasma and peripheral blood mononuclear cells (PBMC) were separated by gradient centrifugation, separated into aliquots, and stored at −80°C. HIV-1/2 status was evaluated in plasma by current serological testing combining enzyme linked immunosorbent assays (ELISAs) and Western blotting. CD4 counts were determined in whole blood using a FACScan flow cytometer (Becton Dickinson). HIV-1 viral load was quantified in plasma by the Amplicor HIV-1 Monitor assay version 1.5 (Roche). Genomic DNA was extracted from PBMC using a QIAamp DNA blood mini kit (Qiagen). A PCR fragment spanning approximately 560 bp of HIV-1 *env* gp41 was amplified from genomic DNA using previously described primers [24]. The PCR products were purified using QIAamp purification columns (Qiagen) and consensus sequences were determined by standard dideoxy terminator sequencing at a local facility. All molecular analyses were performed in a blinded fashion and strict laboratory procedures, including the use of negative controls in all amplification experiments, were applied in order to prevent cross-specimen contaminations. The sequences were conservatively edited using Sequence Scanner version 1.0 (Applied Biosystems), and then aligned and gap stripped with BioEdit version 7.0.5.3 (North Carolina State University). This resulted in a 520 bp consensus sequence alignment corresponding to positions 7849 to 8368 of the HXB2 genome. All sequences were submitted to Genbank (GenBank accession numbers JQ438031-JQ438118).

### Molecular analysis of HIV-1 transmission

A maximum-likelihood phylogenetic tree incorporating gp41 sequences from all individuals was constructed using MEGA5 software [25]. The software applied a general time reversible nucleotide substitution model with a gamma distribution of rates and a proportion of invariant sites as the most suitable genetic substitution model for the calculation of the pairwise distances. Bootstrap values were calculated from 500 replicates; sequences clustering on the same branch with a bootstrap value of 80% or more were considered to be genetically linked [26]–[29]. Because spurious clustering could occur between unrelated sequences of the same rare HIV-1 subtype, an additional distance criterion was applied [26]. To this end, HIV-1 subtypes were first assigned to all individual sequences in the phylogenetic tree by adding reference sequences from the Los Alamos HIV sequence database (http://www.hiv.lanl.gov). Distances between all possible pairs of sequences of the same HIV-1 subtype but from different couples (2082 pairwise distances in total) were then selected to estimate the within-subtype variability of unlinked gp41 sequences of our study population. The average value of this normal distribution minus two times the standard deviation (the lower limit of the 95% confidence interval) was taken as the lower cut-off value for unlinked sequences, for which a value of 0.04 was obtained. Consequently, two unlinked sequences have a 97.5% chance of having a pairwise distance value above this value. Thus, in addition to the phylogenetic criteria, couples' sequences were considered to be genetically linked if their pairwise distance value was below 0.04.

### Interview methods

Male and female partners were interviewed separately about socio-demographics and sexual behavior. Interviews were done by a trained and experienced social assistant who performed oral interviews based on a standardized questionnaire. The social assistant assured that the participants understood all questions, if needed by using different formulations. The social assistant performed the first level of quality control by checking inconsistencies in the reported information during the interview. Importantly, this was done independently for every participant and for every interview; the social assistant did not check for inconsistencies between screening and follow-up interviews or between partners of the same couple. Interview forms were entered into a database twice by two different researchers and checked electronically for errors. Socio-demographic and behavioral data used in this study were extracted from the screening interview. Data not expected to vary in time (e.g. nationality, date of birth, date of marriage, etc.) were checked for consistency with data from the follow-up interview; this is the second level of quality control.

### Epidemiological analysis of HIV-1 transmission

Male and female partners of HIV-1 concordant couples were interviewed about the date of their first positive HIV test, their antiretroviral therapy status and start date (if applicable), and their suspected source of HIV infection. Epidemiological analyses of intracouple transmission and its direction were primarily based on the suspected sources of HIV infection as reported by both partners. Couples for whom information on the suspected source of infection was not available for both partners were excluded from these analyses. We considered couples to report internal HIV transmission if (i) one partner named the other partner as suspected source of infection while the other partner named an outside source and (ii) the reported dates of first positive HIV test and, if applicable, start of antiretroviral therapy did not contradict the reported order of infection events. We considered couples to report external HIV transmission if both partners named an outside source of infection.

### Statistical analysis

Characteristics of male versus female and index versus recipient partners in HIV-1 concordant couples were compared by using non-parametric Mann-Whitney U tests for independent continuous data, Wilcoxon signed rank tests for paired continuous data, or McNemar tests for paired proportional data. Statistical analyses were performed with SPSS version 16.0.

## Results

**Figure 1:**
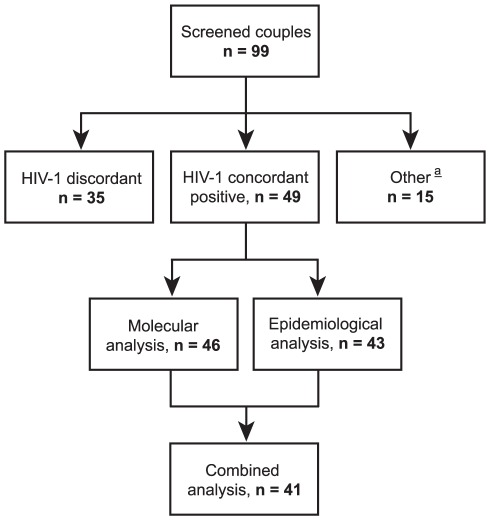
Numbers of couples analyzed in this study. Flow chart showing the numbers of couples that were screened and analyzed in the molecular and epidemiological parts of the study. ^a^These include HIV-2 discordant, HIV-2 concordant positive, and mixed HIV-1/2 concordant positive couples, see text for details.

The numbers of couples that were enrolled and analyzed in the different parts of the study are shown in Figure 1. A total of 99 couples were screened. Forty nine couples (49%) were HIV-1 concordant positive, 35 couples (35%) were HIV-1 discordant, 7 couples (7%) were HIV-2 concordant positive, 4 couples (4%) were HIV-2 discordant, and 4 couples (4%) were mixed HIV-1/2 concordant positive. The demographic, behavioral and clinical characteristics of the 49 HIV-1 concordant positive couples are shown in Table 1. Forty one couples were monogamous consisting of a HIV-1 positive husband with a HIV-1 positive wife. Four partnerships were polygamous each consisting of a HIV-1 positive husband with two HIV-1 positive wives; these were counted as 8 separate couples. Most subjects had a Senegalese nationality and lived in the Dakar metropolitan area, and they reported occupations and levels of education characteristic of those of an African suburban population. No laboratory data were available that would allow to estimate time since primary infection (e.g. prior HIV negative test, incomplete Western blot result suggestive of early infection). A large proportion of subjects were on antiretroviral therapy and those not on therapy had relatively low CD4 counts, suggesting that most subjects were chronically infected. Wives were younger than their husbands, they were less likely on antiretroviral therapy and if not on therapy they had lower viral load levels and higher CD4 counts. No differences were noted in the reported duration of the marriage, the number of sexual contacts per month, or the consistency of condom use between husbands and wives.

**Table 1:** Demographic, behavioural and clinical information of 49 HIV-1 concordant couples included in the study.

	HIV-1 concordant couples (n = 49)		P
	Husbands (n = 45)^a^	Wives (n = 49)	
Age, years	47 (39–52)	33 (29–39)	<0.001^f^
Nationality, n (%)			1.000^g^
Senegal	42 (93)	46 (94)	
Other^b^	3 (7)	3 (6)	
Place of residence, n (%)			0.642^g^
Dakar	38 (84)	43 (88)	
Central-Western region^c^	7 (16)	6 (12)	
Ethnicity			0.549^g^
Woloff	19 (42)	26 (53)	
Poular	16 (36)	17 (35)	
Soninké	6 (13)	2 (4)	
Sérère	2 (4)	2 (4)	
Other^d^	2 (4)	2(4)	
Level of education, n (%)^e^			0.140^g^
None	17 (38)	26 (53)	
Primary school	14 (31)	17 (35)	
Secondary school	10 (22)	5 (10)	
University	4 (9)	1 (2)	
Occupation, n (%)			<0.001^g^
Employee	12 (27)	2 (4)	
Craftsman	14 (31)	5 (10)	
Salesman	10 (22)	9 (18)	
Farmer	1 (2)	0 (0)	
Housewife	0 (0)	32 (65)	
Other	8 (18)	1 (2)	
Duration of marriage, years	8 (4–13)	8 (4–13)	1.000^h^
Number of sexual contacts/month	8 (4–12)	8 (4–12)	0.394^h^
Consistent condom use, n (%)	16 (32)	14 (29)	0.617^i^
Antiretroviral therapy treated, n (%)	30 (71)	23 (47)	0.018^g^
Viral load, log_10_ copies/ml	1.70 (1.70–2.29)	1.70 (1.70–2.24)	0.521^f^
CD4 count, cells/µl	224 (134–344)	302 (227–445)	0.050^f^
Antiretroviral therapy naïve, n (%)	12 (29)	26 (53)	0.018^g^
Viral load, log_10_ copies/ml	5.43 (4.76–5.92)	4.80 (3.85–4.94)	0.028^f^
CD4 count, cells/µl	178 (95–297)	346 (251–686)	0.004^f^

Data are median (interquartile range) values or n (%) when indicated.

a n = 49 for variables for which husbands in 4 polygamous partnerships were asked to report separately for each wife.

b Other nationalities were Mali, Mauretania, Guinea or Guinea-Bissau.

c Region<200 km from Dakar.

d Other ethnicities were Bambara or Diola.

e At least one year of education in the specified grade.

f Mann-Whitney U test was used to compare independent continuous data.

g Chi-square or Fisher's exact test was used to compare independent proportional data.

h Wilcoxon signed rank test was used to compare paired continuous data.

i McNemar test was used to compare paired proportional data.

**Figure 2:**
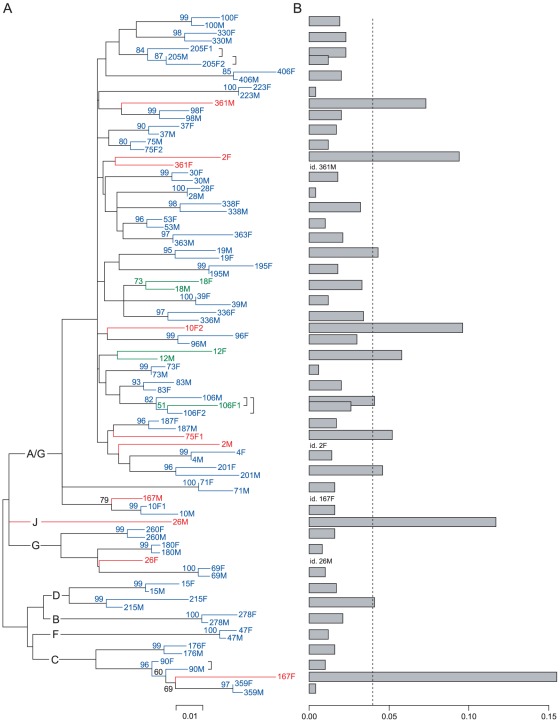
Molecular analysis of HIV-1 transmission in 46 HIV-1 concordant couples. (A) Maximum-likelihood phylogenetic tree. All individual sequences are labeled by their couple number followed by M (male partner) or F (female partner). Female partners in polygamous partnerships are labeled as F1 and F2. Bootstrap values are calculated from 500 replicates; values of 50% or more are shown. Sequences from partners of the same couple are colored in blue if they cluster with a bootstrap value of 80% or more; in green if they cluster with a bootstrap value below 80%; in red if they are on separate branches of the tree. HIV-1 subtypes are shown on the main branches of the tree, HIV-1 reference sequences are omitted. The scale represents the maximum-likelihood genetic distance. (B) Maximum-likelihood genetic distances. Distance values between sequences of male and female partners of the same couple are shown. Bars correspond to the pairs of clustered or non-clustered sequences as they appear in the phylogenetic tree in panel A. Distance values between pairs of non-clustered sequences are shown for only one of the two sequences; the other sequence is labeled “id.” (idem). The dotted line represents the distance cut-off value for linked sequences which is set at 0.04.

We first set out to determine intracouple transmission by conducting a molecular analysis of a 520 bp fragment of the gp41 gene. Gp41 amplification failed for both partners in 3 couples despite repeated PCR attempts with different primer sets. This resulted in a total of 88 gp41 sequences representing 46 HIV-1 concordant couples that were used for the calculation of pairwise genetic distances and the construction of a maximum-likelihood phylogenetic tree (Figure 2). The majority (74%) of the sequences were of subtype A/G (CRF_02), with the remaining sequences belonging to subtype B, C, D, F, G, and J (Figure 2A). Sequences from 37 couples clustered with bootstrap values of 80% or more, three couples showed clustering but with a bootstrap value below 80%, and sequences from 6 couples did not cluster (Figure 2A). Sequences from 35 couples showed pairwise distances below the cut-off value of 0.04, whereas sequences from 11 couples showed pairwise distances above the cut-off (Figure 2B). Together, this resulted in 34 couples with viral sequences clustering with a bootstrap value of at least 80% and a distance value below 0.04; these were conservatively classified as “genetically linked”. Six couples' sequences clustered but without meeting the bootstrap and/or distance criteria; these were classified as “undetermined”. Finally, sequences from 6 couples that did not cluster (and as expected showed distance values above the cut-off value) were classified as “genetically unlinked”.

Next, we investigated HIV-1 transmission by analyzing the interview data. Forty three of 49 couples (88%) reported a suspected source of HIV infection for both partners (Table S1). Six couples (12%) had missing information on the suspected source of infection for either one (5 couples) or both partners (1 couple), these were excluded from the analysis. Among the 43 couples with information on suspected sources of HIV infection for both partners, 36 couples reported internal HIV transmission and 7 couples reported external sources of HIV infection for both partners. None of these couples reported conflicting sources of HIV infection. None of the couples reporting internal HIV transmission provided dates of first positive HIV test and start of antiretroviral therapy that contradicted the reported order of infection events. Only one couple, couple 187, reported conflicting dates of first positive HIV test, however the information provided by the husband did not correspond with his low CD4 count at the time of enrolment (Table S1).

Then, we compared the molecular and epidemiological data for 41 HIV-1 concordant couples with both data available. The molecular and epidemiological data corresponded completely (Table 2). All couples with genetically linked viruses reported internal HIV transmission. All couples with genetically unlinked viruses reported external sources of HIV infection for both partners. Interestingly, all couples with undetermined viral linkage also reported internal HIV transmission.

**Table 2:** Comparison of epidemiological and molecular analyses of HIV-1 transmission for 41 HIV-1 concordant couples.

	Molecular analyses					
	Linked (n = 30)		Undetermined (n = 5)		Unlinked (n = 6)	
Reported source of infection	Husbands	Wives	Husbands	Wives	Husbands	Wives
**Internal transmission (n = 35)**	**30**	**30**	**5**	**5**	**0**	**0**
Spouse in the study	2	28	0	5		
Other or previous spouse	4	1	0	0		
Other stable partner	0	0	1	0		
Occasional sexual relationship	23	1	4	0		
Intravenous drug use	1	0	0	0		
**External sources (n = 6)**	**0**	**0**	**0**	**0**	**6**	**6**
Spouse in the study					0	0
Other or previous spouse					1	5
Other stable partner					0	0
Occasional sexual relationship					5	1
Intravenous drug use					0	0

Data are cross-tabulated numbers of subjects.

For 30 HIV-1 concordant couples with confirmed intracouple transmission by molecular as well as epidemiological methods, we analyzed the reported sources of infection and the direction of the intracouple transmission (Table 2). Twenty eight (93%) of 30 couples reported the husband to be the source of the intracouple transmission. In 23 (82%) of these 28 couples, the husbands reported to have acquired HIV-1 through an occasional sexual relationship. Overall, husbands predominantly reported an occasional sexual relationship as the source of their infection, while for the wives this was almost always the current or previous husband.

Finally, for the 30 confirmed HIV-1 transmission pairs, we compared the reported order of infection events with a paired index/recipient analysis of clinical parameters of HIV infection (Table 3). Index partners reported their first positive HIV test significantly earlier than recipient partners, they were more frequently on antiretroviral therapy and for a significantly longer period of time, and they presented with a significantly lower CD4 count. Kouyos et al recently showed that the proportion of HIV-1 pol ambiguous base pairs can be used as a marker of time since infection [30], and similarly we found that index partners had a significantly higher proportion of gp41 ambiguous base pairs than recipient partners. However, none of these markers are well suited to precisely estimate and compare individual time since infection, especially when subjects are on antiretroviral therapy, and as expected they showed considerable overlap between index and recipient partners. Nevertheless, at the level of our study population, they corroborated the observed patterns of HIV-1 transmission.

**Table 3:** Paired analysis of clinical characteristics of 30 confirmed HIV-1 transmission pairs.

	HIV-1 transmission pairs (n = 30)		P
	Index (n = 30)	Recipients (n = 30)	
Time since first positive HIV test, years	2 (0.5–5)	2 (0.5–3)	0.032^c^
Antiretroviral therapy treated, n (%)	22 (79)	12 (40)	0.007^d^
Time on ART, years			
All couples	1 (0.5–3)	0 (0–1)	0.015^c^
Couples with both partners on ART^a^	3 (2–5)	2 (0.5–5)	0.067^c^
CD4 count, cells/µl			
All couples	233 (169–331)	312 (244–525)	0.011^c^
Couples with both partners on ART^a^	240 (197–511)	302 (230–527)	0.374^c^
Couples with both partners ART-naïve^b^	173 (95–228)	411 (169–711)	0.068^c^
Viral load, log_10_ copies/ml			
All couples	1.75 (1.70–4.26)	3.40 (1.70–4.86)	0.131^c^
Couples with both partners on ART^a^	1.70 (1.70–1.92)	1.70 (1.70–2.42)	0.593^c^
Couples with both partners ART-naïve^b^	5.87 (5.07–5.95)	4.85 (4.43–5.45)	0.144^c^
Ambiguous base pairs, %			
All couples	2.60 (1.54–3.65)	1.73 (0.82–2.69)	0.038^c^
Couples with both partners on ART^a^	2.31 (1.35–3.65)	1.54 (1.15–2.50)	0.374^c^
Couples with both partners ART-naïve^b^	1.64 (0.53–6.49)	2.60 (0.82–4.24)	0.715^c^

Data are median (interquartile range) values or n (%) when indicated.

a n = 9 couples;

b n = 4 couples;

c Wilcoxon signed rank test was used to compare paired continuous data.

d McNemar test was used to compare paired proportional data.

## Discussion

In this study, we provide conservative molecular evidence for internal HIV-1 transmission in a large proportion of Senegalese HIV-1 concordant couples. The majority of these couples reported the male partner as the source of the intracouple transmission, who in turn most frequently reported to have acquired HIV through an occasional sexual relationship. These data suggest that HIV-1 is frequently transmitted within married couples in Senegal with the husband as the most likely index partner. Our findings are in line with previous reports showing that married women in Africa have a high risk for acquiring HIV as a result of the extramarital relationships of their husbands [8], [15], [31], [32]. Our data also corroborate studies proposing concurrency as an important risk factor for onward HIV-1 transmission to the stable partner [13]–[17]. Indeed, in our study, several husbands in linked couples who reported an occasional sexual relationship as source of infection had their first positive HIV-1 test many years after marriage (Table S1), suggesting that their occasional relationships concurred with marriage. Under such conditions, HIV can rapidly spread from the occasional relationship to the wife as a result of the highly contagious acute infection of the husband [33], [34], as well as from the often undiagnosed and/or undisclosed HIV status of the husband during this period [35], [36].

The high proportion of linked transmissions (74%) in our cohort is in agreement with previous studies of HIV-1 transmission in African married couples. Two large HIV prevention trials in cohorts of HIV-1 discordant couples found genetically linked transmissions in 72% and 76% of the cases, respectively [37], [38]. A prospective study of HIV-1 discordant couples from Zambia found 87% of linked transmissions [26]. Nevertheless, considerable numbers of HIV-1 acquisitions in these and our studies were unlinked to the partner's virus, thus emphasizing the need for molecular confirmation of HIV-1 transmission before embarking on clinical trials or studies of risk of HIV-1 transmission.

Our data are not in agreement with recent meta-analyses of HIV-1 discordant couple cohorts estimating large proportions of wife-to-husband transmissions in African married couples [9]–[11]. A possible reason for this discrepancy could be the existence of an inclusion bias in HIV-1 discordant couple cohorts resulting in an overrepresentation of couples with HIV-1 infected wives. Indeed, in our Senegalese cohort we also counted more HIV-1 discordant couples with HIV-1 infected wives than with HIV-1 infected husbands (author's unpublished data and ref. [39]). In fact, we noted that HIV-1 infected husbands frequently declined to participate because of fear of disclosing their HIV status to their wives (author's unpublished data). HIV-1 infected wives felt no stigma preventing them from participating because they often had an already disclosed HIV-1 infection originating from a previous marriage. In addition, HIV-1 discordant couples with HIV-1 infected husbands probably evolve more rapidly to a HIV-1 concordant status, exactly because of the husbands' frequent undisclosed and acute infection status, and as such they are not available for inclusion in HIV-1 discordant couple cohorts. At least the latter factor can be expected not to influence HIV-1 concordant couple cohorts, given that they can be enrolled any time after intracouple transmission has taken place. It is possible that HIV-1 infected men who are the index partners in HIV-1 concordant couples feel similar stigma preventing them from participating like those in HIV-1 discordant couples. However we expect this to be less given that both partners are now HIV-1 positive. In any case, such bias would not have influenced our conclusions as it would have led to an underestimation of the frequency of husband-to-wife transmissions in our Senegalese study population. Together, HIV-1 concordant couple cohorts with molecularly confirmed internal transmission probably allow a more trustworthy estimation of the relative frequencies of male-to-female and female-to-male transmission than HIV-1 discordant couple cohorts.

An important limitation of HIV-1 concordant couple studies however is that they have to rely on interview data to determine the direction of the intracouple transmission. To date, the order of infection events cannot easily be deduced from viral sequence information [37], [40], [41]. Interview data may be subject to reporting error and bias, especially when they concern sensitive matter like sexual behavior. Indeed, one wonders to what extent the high proportion of reported male-to-female transmissions in our cohort could reflect women's resistance to report extramarital relationships. On the other hand, several observations support the reliability of the data that were used in our study. First, the interview data corresponded completely with the results of the molecular analyses: all couples with genetically linked viruses reported internal HIV transmission and all couples with genetically unlinked viruses reported external sources of HIV infection. Second, none of the couples reported conflicting sources of HIV infection for both partners. And finally, a number of clinical parameters of HIV-1 infection like the time since self-reported first positive HIV test, antiretroviral therapy status and duration, CD4 count, and the proportion gp41 ambiguous base pairs corroborated the reported order of infection events. Couples for whom information on the suspected source of infection was not available for both partners were excluded from the analyses. However it is unlikely that this has influenced our conclusions given that it affected only 6 couples and that their partially reported sources of infection were very comparable to the total study population (Table S1).

The application of phylogenetic analysis to confirm HIV-1 transmission events is well established, and it is frequently used for providing evidence in legal cases [42], [43], HIV prevention trials [37], [38], or specific epidemiological or molecular studies [29], [44], [45]. The choice of an appropriate HIV-1 gene is crucial as it should match the genetic variability of the target population [46]–[48]. For instance, the rapid genetic diversification of the variable *env* gp120 V2–V3 region makes it ideal for studying recent transmission events. On the other hand, the low genetic variability of HIV-1 *pol* makes it more suitable for establishing older transmissions. Assuming that our Senegalese cohort of HIV-1 concordant couples contained recent as well as older HIV-1 transmissions, we decided to use *env* gp41, a gene with a genetic variability in between that of *env* gp120 and *pol*. Previous studies successfully used gp41 in HIV-1 subtyping and linkage analyses of similarly diverse datasets [26], [49], [50]. The use of gp41 sequences in our study population showed high specificity and sensitivity relative to the interview data. Our assays lacked sensitivity to prove genetic linkage for 6 couples, 5 of whom reported internal HIV-1 transmission. These couples yielded undetermined molecular results, i.e. the gp41 sequences clustered in the phylogenetic tree but without meeting the conservative bootstrap and distance criteria. Relaxation of the bootstrap and distance criteria could increase the sensitivity without affecting the specificity. Alternatively, more conserved HIV-1 *gag* or *pol* genes or the use of clonal or deep sequencing techniques could help establish genetic linkage in these couples, but this was beyond the scope of this study.

Phylogenetic analyses cannot exclude that two linked sequences are connected through a third intermediate sequence not included in the analysis. This possibility could frequently occur in populations of e.g. men who have sex with men where promiscuous behavior has been shown to result in large clusters of linked viruses with uncertainty of the origins and directionality of the underlying transmissions [51], [52]. However, such interconnectivity is unlikely in our study population of well-defined heterosexual partnerships originating from a large metropolitan area and characterized by much less promiscuous behavior than men who have sex with men. Therefore, the majority of the observed phylogenetic clusters in our study likely represent genuine intracouple transmissions. Another consideration that should be taken into account in phylogenetic studies is HIV-1 superinfection, i.e. the infection of an already HIV-1 positive individual with a second HIV-1 strain [53]. For instance, it could be possible that for some linked couples in our study both partners initially acquired HIV-1 from outside their relationship and that a subsequent superinfection from one partner to the other was picked up by our assays as a linked transmission. Detection of HIV-1 superinfection can be complex and requires clonal or deep sequencing techniques which was beyond the scope of our study. Nevertheless, given that the genetic analyses in our study were completely supported by the interview data, and that frequencies of HIV-1 superinfection are considered to be low in low-risk populations with low background HIV-1 prevalences [53], superinfection likely has not confounded our observations much.

The majority of the couples enrolled in our study lived in the Dakar metropolitan area, and they reported a wide range of occupations and levels of education characteristic of those of an African suburban population. Therefore, we should be careful with extrapolating the observed patterns of HIV-1 transmission in this study population to populations living in more rural areas or in other African countries. Future studies should investigate HIV-1 transmission in HIV-1 concordant couple cohorts enrolled from other African populations with different socio-epidemiological factors.

In summary, we found that a majority of HIV-1 concordant couples in Dakar, Senegal showed genetically linked HIV-1 transmission with the husband as likely index partner. Our data emphasize the risk of married women for acquiring HIV-1 as a result of the occasional sexual relationships of their husbands. Understanding the origins and dynamics of HIV-1 infection in married couples in Africa will be crucial for the future planning of successful HIV prevention programs.

## Supporting Information

Table S1 **Epidemiological analyses of HIV-1 transmission for 49 HIV-1 concordant couples.**
(DOC)
