# Supplementary material for: Molecular Epidemiology of HIV-1 Transmission in a Cohort of HIV-1 Concordant Heterosexual Couples from Dakar, Senegal
Source: PLoS One. 2012 May 17;7(5):e37402. doi: 10.1371/journal.pone.0037402 (PMC3355130; doi:10.1371/journal.pone.0037402)
Supplement: Table S1 — Epidemiological analyses of HIV-1 transmission for 49 HIV-1 concordant couples. (DOC) [file pone.0037402.s001.doc]

**Table S1. Epidemiological analyses of HIV-1 transmission for 49 HIV-1 concordant** couples.

|  | **Husband** | | | |  | **Wife** | | | |  | **Conclusion** | |
| --- | --- | --- | --- | --- | --- | --- | --- | --- | --- | --- | --- | --- |
| **Couple number** | **Start relation** | **First  HIV+ test** | **Start treatment** | **HIV source** |  | **Start relation** | **First  HIV+ test** | **Start treatment** | **HIV source** |  | **Transmission** | **Direction** |
| **Internal (n=36)** |  |  |  |  |  |  |  |  |  |  |  |  |
| 4 | 2001 | N/A | 2000 | First wife |  | 2001 | 2003 | None | Husband |  | Internal | Husband to wife |
| 10a | 1996 | 2001 | 2002 | Occasional relation |  | 1996 | 2001 | 2001 | Husband |  | Internal | Husband to wife |
| 12 | 1994 | 1997 | 1998 | Occasional relation |  | 1994 | 1997 | 1998 | Husband |  | Internal | Husband to wife |
| 18 | 1994 | 1996 | 2002 | Occasional relation |  | 1994 | 1996 | 2003 | Husband |  | Internal | Husband to wife |
| 19 | 1995 | 2000 | 2000 | Other partner |  | 1995 | 2000 | 2004 | Husband |  | Internal | Husband to wife |
| 28 | 1992 | 2004 | 2004 | Other wife |  | 1992 | 2004 | None | Husband |  | Internal | Husband to wife |
| 30 | 2001 | 2004 | 2004 | Occasional relation |  | 2001 | 2004 | None | Husband |  | Internal | Husband to wife |
| 32 | 1999 | 2003 | 2003 | First wife |  | 1999 | 2003 | None | Husband |  | Internal | Husband to wife |
| 39 | 1996 | 2004 | None | Wife |  | 1996 | 2003 | 2004 | First husband |  | Internal | Wife to husband |
| 47 | 2001 | 2004 | 2004 | Wife |  | 2001 | 2004 | 2004 | Occasional relation |  | Internal | Wife to husband |
| 53 | 1993 | 2001 | 2002 | Occasional relation |  | 1993 | 2001 | None | Husband |  | Internal | Husband to wife |
| 69 | 2002 | 2003 | 2003 | Occasional relation |  | 2002 | 2004 | 2004 | Husband |  | Internal | Husband to wife |
| 73 | 1998 | 2000 | 2002 | Occasional relation |  | 1998 | 2002 | 2002 | Husband |  | Internal | Husband to wife |
| 75b | 1994 | 2000 | None | Occasional relation |  | 1994 | 2002 | None | Husband |  | Internal | Husband to wife |
| 83 | 1975 | N/A | N/A | Occasional relation |  | 1975 | 2004 | None | Husband |  | Internal | Husband to wife |
| 90 | 1999 | 2002 | 2003 | Occasional relation |  | 1999 | 2002 | 2004 | Husband |  | Internal | Husband to wife |
| 96 | 1976 | 2004 | 2004 | Occasional relation |  | 1976 | 2004 | None | Husband |  | Internal | Husband to wife |
| 98 | 1998 | 2000 | 2000 | Occasional relation |  | 1998 | 2002 | None | Husband |  | Internal | Husband to wife |
| 100 | 2001 | 2002 | 2002 | Occasional relation |  | 2001 | 2002 | None | Husband |  | Internal | Husband to wife |
| 106a | 1997 | 1997 | 2005 | Occasional relation |  | 1997 | 2005 | 2005 | Husband |  | Internal | Husband to wife |
| 106b | 1980 | 1997 | 2005 | Occasional relation |  | 1980 | N/A | 2005 | Husband |  | Internal | Husband to wife |
| 176 | 1977 | 2004 | None | Occasional relation |  | 1977 | 2004 | None | Husband |  | Internal | Husband to wife |
| 180 | 1999 | 2001 | 2002 | Occasional relation |  | 1999 | 2003 | None | Husband |  | Internal | Husband to wife |
| 187 | 1995 | 2005^a^ | None | Occasional relation |  | 1995 | 2001 | 2005 | Husband |  | Internal | Husband to wife |
| 195 | 2003 | 2005 | 2005 | Occasional relation |  | 2003 | 2005 | None | Husband |  | Internal | Husband to wife |

Couple numbers with a or b at the end designate polygamous partnerships split into separate couples: a, with the first wife; b, with the second wife. ^a^HIV infection likely occurred earlier as this subject presented with a CD4 count of 119 cells/µl in January 2006. N/A, not available.

**Table S**1. Continued.

|  | **Husband** | | | |  | **Wife** | | | |  | **Conclusion** | |
| --- | --- | --- | --- | --- | --- | --- | --- | --- | --- | --- | --- | --- |
| **Couple number** | **Start relation** | **First  HIV+ test** | **Start treatment** | **HIV source** |  | **Start relation** | **First  HIV+ test** | **Start treatment** | **HIV source** |  | **Transmission** | **Direction** |
| 205a | 1986 | 1999 | 1999 | Occasional relation |  | 1986 | 2000 | 2001 | Husband |  | Internal | Husband to wife |
| 205b | 1999 | 1999 | 1999 | Occasional relation |  | 1999 | 2001 | 2001 | Husband |  | Internal | Husband to wife |
| 215 | 1993 | 2004 | 2005 | Occasional relation |  | 1993 | 2005 | None | Husband |  | Internal | Husband to wife |
| 223 | 1993 | 2001 | 2001 | First wife |  | 1993 | 2002 | 2002 | Husband |  | Internal | Husband to wife |
| 260 | 1996 | 2005 | None | Occasional relation |  | 1996 | 2005 | 2005 | Husband |  | Internal | Husband to wife |
| 278 | 1974 | 2005 | 2005 | Other wife |  | 1974 | 2005 | None | Husband |  | Internal | Husband to wife |
| 336 | 1982 | 2005 | None | Drug use |  | 1982 | 2005 | None | Husband |  | Internal | Husband to wife |
| 338 | 2000 | 2004 | None | Occasional relation |  | 2000 | 2004 | None | Husband |  | Internal | Husband to wife |
| 359 | 2001 | 2005 | None | Occasional relation |  | 2001 | 2005 | 2005 | Husband |  | Internal | Husband to wife |
| 363 | 1979 | 1999 | 2001 | Occasional relation |  | 1979 | 2004 | None | Husband |  | Internal | Husband to wife |
| 406 | 1996 | 2006 | 2006 | Occasional relation |  | 1996 | 2006 | None | Husband |  | Internal | Husband to wife |
|  |  |  |  |  |  |  |  |  |  |  |  |  |
| **External (n=7)** |  |  |  |  |  |  |  |  |  |  |  |  |
| 2b | 2005 | 1997 | 2000 | Other Wife |  | 2005 | 2004 | None | First husband |  | External | N/A |
| 10b | 2004 | 2001 | 2002 | Occasional relation |  | 2004 | N/A | None | First husband |  | External | N/A |
| 26 | 2004 | 2002 | None | Occasional relation |  | 2004 | 2000 | 2000 | First husband |  | External | N/A |
| 61 | 2000 | 1991 | None | Occasional relation |  | 2000 | 1996 | 1996 | First husband |  | External | N/A |
| 75a | 2000 | 2000 | None | Occasional relation |  | 2000 | 2000 | 2000 | First husband |  | External | N/A |
| 167 | 2004 | 1998 | 2000 | Occasional relation |  | 2004 | 2001 | 2004 | First husband |  | External | N/A |
| 361 | 2005 | 2002 | 2002 | Occasional relation |  | 2005 | 1997 | 1999 | Occasional relation |  | External | N/A |
|  |  |  |  |  |  |  |  |  |  |  |  |  |
| **Unknown (n=6)** |  |  |  |  |  |  |  |  |  |  |  |  |
| 15 | N/A | N/A | N/A | Occasional relation |  | N/A | N/A | None | N/A |  | N/A | N/A |
| 37 | 1980 | 2003 | 2002 | N/A |  | 1980 | 2003 | None | Husband |  | N/A | N/A |
| 49 | 2000 | 2004 | None | N/A |  | 2000 | 2004 | None | N/A |  | N/A | N/A |
| 71 | 1997 | 2004 | 2004 | N/A |  | 1997 | 2004 | None | Husband |  | N/A | N/A |
| 201 | 1996 | 2005 | None | N/A |  | 1996 | 2005 | None | Husband |  | N/A | N/A |
| 330 | 2003 | 2005 | None | N/A |  | 2003 | 2005 | 2005 | Husband |  | N/A | N/A |

Couple numbers with a or b at the end designate polygamous partnerships split into separate couples: a, with the first wife; b, with the second wife. N/A, not available.
